# Preoperative nTMS and Intraoperative Neurophysiology - A Comparative Analysis in Patients With Motor-Eloquent Glioma

**DOI:** 10.3389/fonc.2021.676626

**Published:** 2021-05-21

**Authors:** Tizian Rosenstock, Mehmet Salih Tuncer, Max Richard Münch, Peter Vajkoczy, Thomas Picht, Katharina Faust

**Affiliations:** ^1^ Department of Neurosurgery, Charité – Universitätsmedizin Berlin, corporate member of Freie Universität Berlin and Humboldt-Universität zu Berlin, Berlin, Germany; ^2^ Berlin Institute of Health at Charité – Universitätsmedizin Berlin, Biomedical Innovation Academy, Berlin, Germany; ^3^ Cluster of Excellence: “Matters of Activity. Image Space Material”, Humboldt University, Berlin, Germany

**Keywords:** navigated transcranial magnetic stimulation (nTMS), brain tumor surgery, glioma, motor outcome, diffusion tensor imaging, intraoperative neurophysiological monitoring (IOM), motor-evoked potential (MEP), subcortical stimulation

## Abstract

**Background:**

The resection of a motor-eloquent glioma should be guided by intraoperative neurophysiological monitoring (IOM) but its interpretation is often difficult and may (unnecessarily) lead to subtotal resection. Navigated transcranial magnetic stimulation (nTMS) combined with diffusion-tensor-imaging (DTI) is able to stratify patients with motor-eloquent lesion preoperatively into high- and low-risk cases with respect to a new motor deficit.

**Objective:**

To analyze to what extent preoperative nTMS motor risk stratification can improve the interpretation of IOM phenomena.

**Methods:**

In this monocentric observational study, nTMS motor mapping with DTI fiber tracking of the corticospinal tract was performed before IOM-guided surgery for motor-eloquent gliomas in a prospectively collected cohort from January 2017 to October 2020. Descriptive analyses were performed considering nTMS data (motor cortex infiltration, resting motor threshold (RMT), motor evoked potential (MEP) amplitude, latency) and IOM data (transcranial MEP monitoring, intensity of monopolar subcortical stimulation (SCS), somatosensory evoked potentials) to examine the association with the postoperative motor outcome (assessed at day of discharge and at 3 months).

**Results:**

Thirty-seven (56.1%) of 66 patients (27 female) with a median age of 48 years had tumors located in the right hemisphere, with glioblastoma being the most common diagnosis with 39 cases (59.1%). Three patients (4.9%) had a new motor deficit that recovered partially within 3 months and 6 patients had a persistent deterioration (9.8%). The more risk factors of the nTMS risk stratification model (motor cortex infiltration, tumor-tract distance (TTD) ≤8mm, RMT_ratio_ <90%/>110%) were detected, the higher was the risk for developing a new postoperative motor deficit, whereas no patient with a TTD >8mm deteriorated. Irreversible MEP amplitude decrease >50% was associated with worse motor outcome in all patients, while a MEP amplitude decrease ≤50% or lower SCS intensities ≤4mA were particularly correlated with a postoperative worsened motor status in nTMS-stratified high-risk cases. No patient had postoperative deterioration of motor function (except one with partial recovery) when intraoperative MEPs remained stable or showed only reversible alterations.

**Conclusions:**

The preoperative nTMS-based risk assessment can help to interpret ambiguous IOM phenomena (such as irreversible MEP amplitude decrease ≤50%) and adjustment of SCS stimulation intensity.

## Introduction

When resecting a glioma, a gross total resection (GTR) is always aimed for since the extent of resection (EOR) is positively correlated with (progression free) survival (1, 2). A large multicenter trial demonstrated a benefit of supramarginal resections (= resection of both contrast-enhanced and noncontrast-enhanced tumor parts) especially for patients with Isocitrate dehydrogenase (IDH)-wild-type glioblastoma (3). However, it is important to note that patients with eloquently located brain lesions require careful consideration of tumor resection versus functional preservation, as new functional deficits lead not only to reduced quality of life but also to reduced survival (4).

The gold standard for surgical treatment of motor-eloquent brain lesions is resection guided by intraoperative neurophysiological monitoring (IOM) (5). Yet, different techniques, stimulation intensities, concepts, and warning signs can be found in the literature, making interpretation of IOM phenomena difficult (6, 7). In addition, there are reports of false-positive and false-negative monitoring phenomena affecting intraoperative motor outcome prediction as well (8–10). Navigated transcranial magnetic stimulation (nTMS) has been established as a reliable preoperative motor mapping method with very high concordance to direct cortical stimulation (11, 12). A meta-analysis demonstrated the clinical benefit of nTMS motor mapping and showed improvement in motor outcome and extent of resection (13). We developed a risk stratification model based on nTMS and tractography data with which patients with motor-eloquent tumor can be used to stratify into low and high-risk cases (14). High-risk cases (with higher likelihood of postoperative motor deterioration) are characterized by 3 risk parameters: 1.) nTMS-verified motor cortex infiltration, 2.) tumor distance to the diffusion tensor imaging (DTI)-derived corticospinal tract (CST) ≤8mm or 3.) a ratio <90%/>110% of the resting motor threshold (RMT) of both hemispheres (indicating individual and interhemispheric excitability).

A comparison between preoperative nTMS motor mapping/tractography and phenomena of IOM has not been done so far. One can hypothesize that preoperative nTMS assessment can support the interpretation of ambiguous IOM changes (such as reversible MEP amplitude alterations or irreversible MEP amplitude decrease ≤50%) that might be examined more meticulously and carefully in high-risk cases. The aim of this study was to investigate the extent to which preoperative nTMS motor risk stratification and diffusion analysis can help relate ambiguous IOM phenomena to surgical outcome and therefore improve IOM interpretation.

## Materials and Methods

### Study Design

**Figure 1:**
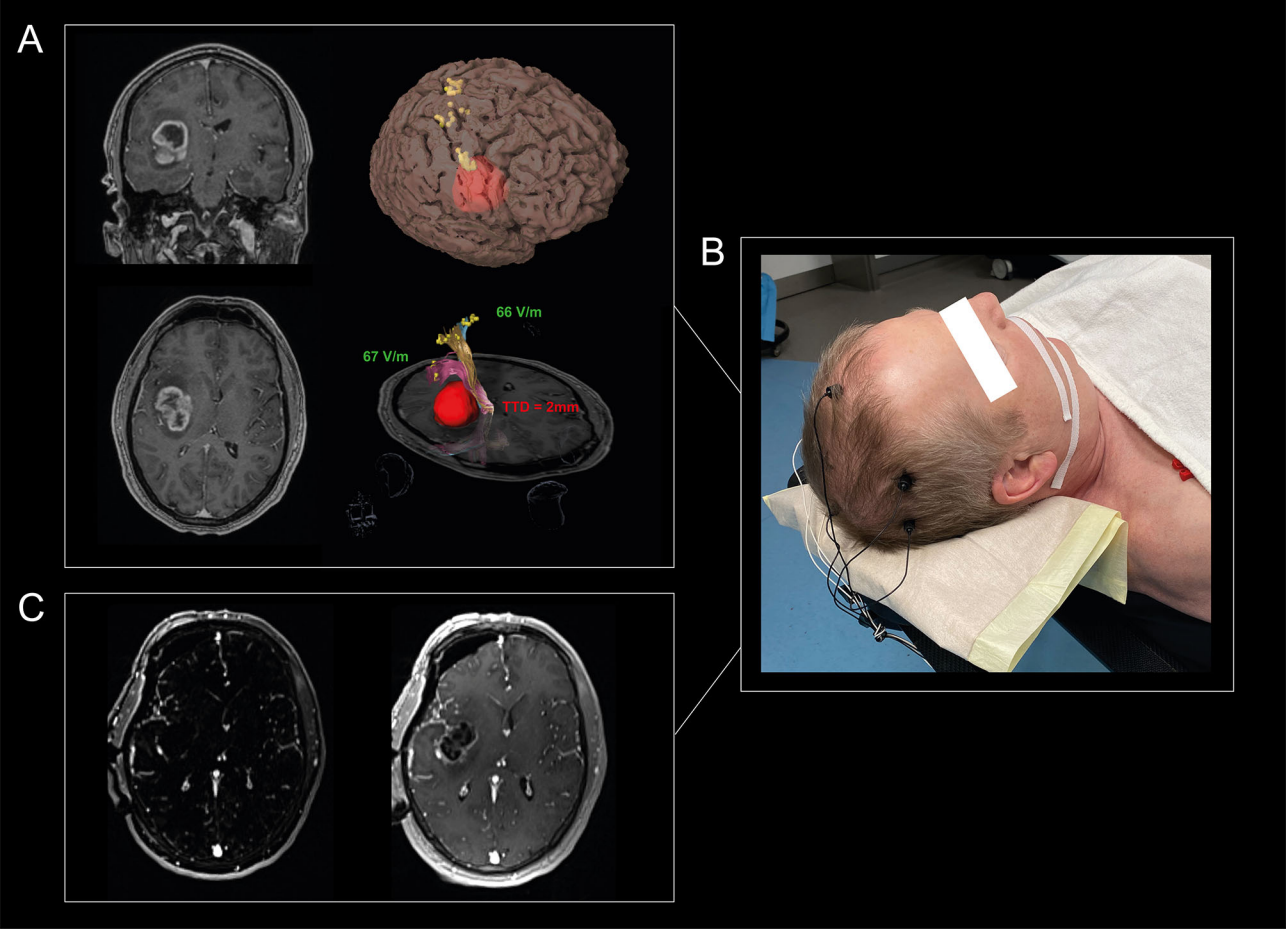
Visualization of our workflow. A 65-year-old man suffered from headache and personality changes. Cerebral MRI showed an insular, contrast-enhancing tumor in the right hemisphere **(A)**. An nTMS motor mapping was performed for the upper extremity, lower extremity, and facial muscles to define the individual cortical motor representation and to investigate the individual excitability level of the patients, which showed a normal RMT_ratio_ between 90% and 110%. Standardized nTMS-based tractography revealed a TTD of 2 mm, so that a total of 1 of 3 risk factors was detectable. Tumor resection was performed using MEPs, SSEPs, and SCS, with SCS guided by the mapping suction probe at a minimum intensity of 2mA. The MEPs showed changes two times, so resection was paused each time and irrigated with papaverine until the MEPs recovered **(B)**. MRI resection control showed a good result with no evidence of residual tumor **(C)**. The patient did not suffer a new motor deficit.

Retrospective analysis of a prospectively collected cohort from 01/2017 to 10/2020 was performed in accordance with the STROBE-Guidelines (15) and the ethical standards of the Declaration of Helsinki. The Ethics Commission approved the study (#EA1/016/19) and the patients provided written informed consent. Our workflow is exemplarily shown in **Figure 1**.

### Patients

Sixty-six patients (age ≥ 18 years) with a motor-eloquent glioma (affecting the motor cortex and/or the CST) who received preoperative nTMS motor mapping and underwent IOM-guided resection were included. Intracranial implants are exemplary contraindications for nTMS (16), however no patient had to be excluded. Patient characteristics such as age, sex and the Karnofsky Performance Scale (17) were assessed in a purpose made database. The motor status was assessed preoperatively, on the day of discharge and after 3 months by the attending neurosurgeon according to the British Medical Research Council [BMRC]) (18) (a scale ranging between 0-5 where BMRC grade 5 represents full strength and 0 representing no muscle activation). The motor outcome was defined as worsened or not worsened, with motor worsening defined as decreased postoperative muscle strength compared with preoperative status. Tumor grading (according to WHO classification of 2016) was performed by the department of neuropathology (19).

### MR Imaging

The MRI scans were performed on a 3 Tesla MRI scanner (Siemens 3T Skyra system, Erlangen, Germany), with technical details published elsewhere (20). A contrast-enhanced 3D gradient echo sequence with a slice thickness of 1mm was used as reference sequence for the nTMS mapping. Preoperative tumor volume was calculated by tumor segmentation with the clinical planning software Elements (Brainlab AG, Munich, Germany) using the T1-weighted sequences with contrast agent in contrast-enhanced tumors and fluid-attenuated inversion recovery (FLAIR) sequences in cases without contrast enhancement. In each patient, DTI sequences were acquired for fiber tracking and analysis of diffusion tensor-based metrics (2-mm isotropic resolution; TR/TE 7500/95ms; 1 shell b-value = 1300 s/mm^2^ with 60 directions per shell) with details published previously (20). The postoperative MRI scan (acquired within 72h postoperatively) was carefully evaluated for ischemia and residual tumor tissue by an interdisciplinary board of neurosurgeons and neuroradiologists based on diffusion weighted imaging (DWI) sequences for ischemia, subtraction sequences (T1 with contrast agent subtracted by T1) for contrast agent enhancing tumors or otherwise based on the FLAIR sequence. The EOR was defined as follows: GTR: complete removal, subtotal resection: residual tumor volume ≤ 15ml and partial resection: > 15ml (21).

### nTMS Mapping

An nTMS motor mapping (NBS 5.1; Nexstim, Helsinki, Finland) was performed in all patients for both hemispheres in accordance with the consensus protocol of an international expert panel (22). Based on the exact localization of the coil and the respective stimulation intensity (measured in V/m as the resulting electric field strength), we performed somatotopic mapping for the following muscles:

- upper extremity: abductor pollicis brevis, first digital interosseus and extensor carpi radialis
- lower extremity: tibialis anterior and abductor hallucis brevis.

A stimulation site was considered positive if the amplitude of the resulting motor evoked potential (MEP) was at least 50µV. The mapping data could also be visualized intraoperatively by implementation into the neuronavigation system so that the motor cortex could be identified. The RMT of each hemisphere (RMT_tumor_, RMT_healthy_) and the ratio (RMT_ratio_ = RMT_tumor_ divided by RMT_healthy_) as surrogate parameters for the cortical excitability were determined based on an adaptive threshold-hunting algorithm (23). We verified whether the tumor infiltrated the gyrus which was identified as motor cortex (= in which MEPs were elicited) on the basis of a 3D MRI reconstruction.

### Tractography

The data of the nTMS motor mapping were then imported *via* DICOM format into our planning software Elements (Brainlab AG, Munich, Germany), followed by image fusion with the MRI data and cranial distortion correction to optimize the planning accuracy (24). Deterministic “*fiber assignment by continuous tracking*” and “*tensor deflection*” algorithms were used to visualize the CST for the upper and the lower extremity in a highly reliable and user-independent manner. We combined an anatomically seeded region of interest in the anterior-inferior pontine level with the nTMS stimulation points as seeding regions (20, 25). Intraoperative validation of the nTMS-based tracking algorithm showed to be superior to other algorithms, specifically visualizing peritumoral tracts while avoiding aberrant fibers (25–28). Finally, the minimum distance between the CST and the tumor was measured and the mean as well as the peritumoral diffusion values fractional anisotropy (FA) and apparent diffusion coefficient (ADC) were calculated (FA_avg_ and the ADC_avg_ [mm^2^/s] of the entire tracts, FA_tumor_ and ADC_tumor_ at the tumor level).

### Intraoperative Neurophysiological Monitoring

All patients received standard anesthesia (total venous anesthesia and short-acting relaxants for intubation) with weight-adjusted use of propofol, fentanyl and remifentanil. IOM was applied in all cases with the ISIS system (Inomed, Emmendingen, Germany), with the treating neurosurgeon deciding individually on the monitoring techniques used:

-MEPs (n = 61) (train of 5, pulse duration: 0.2 msec, interstimulus interval: 2 msec) with corkscrew electrodes placed at C_3_ and C_4_ according to the 10–20 electroencephalography (EEG) system (29)
-continuous subcortical stimulation (SCS) (n = 53) using a monopolar mapping suction probe at a frequency of 2 Hz with a stimulation intensity of 10-15 mA initially, which was reduced depending on the individual case (7)
-recordings of the somatosensory evoked potential (SSEP) (n = 29) of the medianus and posterior tibial nerve with computer-assisted averaging and corkscrew electrodes additionally placed at F_z_, C_z_’, C_3_’ and C_4_’ (stimulating intensities: 15-25 mA; current pulses: 0.2 msec; filter settings: 7 Hz–5 kHz) (30)

MEPs were recorded by pairs of needle electrodes inserted in the same muscles as mentioned above. Continuous EEG monitoring (using the corkscrew scalp electrodes) was performed to detect seizures and to keep the depth of anesthesia constant.

A decrease in MEP or SSEP amplitude >50% was considered a warning sign and the resection was paused immediately. Nonsurgical reasons for the decrease such as hypotension, altered anesthetic regimen (anesthetic use/ventilation parameters), or hypothermia were immediately evaluated (7, 31). Papaverine was applied aiming for full recovery of MEP and SSEP amplitude. It was recorded whether an amplitude decrease was transient or permanent and whether the permanent decrease was ≤50% or >50%. Resection was terminated when there was an irreversible MEP decrease >50% or subcortical stimulation indicated a very close location of the CST. We documented the (minimal) stimulation intensity of the mapping suction probe.

### Statistical Analysis

Statistics were performed with IBM SPSS Statistics 25.0 (IBM Corp., Armonk, N.Y., USA). A two-sided statistical significance level of α = 0.05 was used. Descriptive analyses were performed by reporting the mean and standard deviation (SD) for normally distributed parameters or the median and interquartile range (IQR) otherwise. Standardized mean differences (SMD) and the contingency coefficient (cc) were calculated to indicate sample size independent magnitude of group differences. The Pearson’s Chi-Square Test, two sample t-Test or Mann-Whitney-U Test were used depending on the scaling and distribution of the variables. Correlation analyses of metric parameters were performed by calculating Pearson correlation coefficient r.

## Results

### Patients Sample

This consecutive cohort consisted of 66 patients with a median age of 48 years whose characteristics are detailed in **Table 1**. Most patients suffered from a glioblastoma (59.1% of cases). The preoperative clinical examination revealed a motor deficit in 12 patients (18.2%). A new/an agravated postoperative paresis was found in 11 patients (16.7%) at the day of discharge. One patient (1.5%) showed an improvement in motor function postoperatively. A persistent paresis at 3-month follow-up occurred in 6 patients (9.1%) and 3 patients showed a partial recovery (4.5%) with a BMRC grade ≥ 3. The proportion of patients who developed a new postoperative motor deficit was the same in patients with preoperative paresis (16.7%) and without preoperative paresis (16.7%). Five patients (7.6%) were lost to follow-up for the following reasons: 3 patients with tumor progression, 1 patient died, and 1 patient moved to another city.

**Table 1:** Patient sample.

	n = 66
**Age** in years, median (IQR)	48y (28)
**Sex**	
Female	27 (40.9%)
Male	39 (59.1%)
**KPS**, median (IQR)	90 (13)
**BMRC_preop_**.	
0-3	5 (7.6%)
4	7 (10.6%)
5	54 (81.8%)
**Hemisphere**	
R	37 (56.1%)
L	29 (43.9%)
**Tumor Location**	
Frontal	23 (34.8%)
Parietal	18 (27.3%)
Temporo-insular	22 (33.3%)
Multilocular	3 (4.5%)
**Histology**	
WHO II°	10 (15.2%)
WHO III°	17 (25.8%)
WHO IV°	39 (59.1%)
**Tumor Recurrency**	22 (33.3%)
**Tumor Volume** in ml, median (IQR)	23.35 (32.58)
**Edema** within CST	36 (54.5%)
**IOM**	
MEP	61 (92.4%)
SSEP	29 (43.9%)
SCS	53 (80.3%)
**Motor Outcome**	
Day of Discharge	
Worsening	11 (16.7%)
No Worsening	55 (83.3%)
3 Months postop.	
missings^1^	5 (7.6%)
Persistent Worsening	6 (9.1%)
Partial Recovery	3 (4.5%)
No Worsening	52 (78.8%)
**Extent of Resection**	
GTR	46 (65.7%)
STR	20 (28.6%)
PR	4 (5.7%)

^1^3 patients with tumor progression, 1 patient died, and 1 patient lived in another city; KPS, Karnofsky Performance Scale; BMRC, motor status according to the British Medical Research Counsil; CST, corticospinal tract; MEP, motor evoked potentials; SSEP, somatosensory evoked potentials; SCS, subcortical stimulation; GTR, gross total resection; STR, subtotal resection; PR, partial resection.

Postoperative ischemia was detected in 20 patients (30.3%), of whom 3 (4.5%) were subcortically located in the course of CST and one (1.5%) was partially located in the motor cortex (**Table 1**). Subcortical ischemic injury resulted in a persistent motor deficit in all 3 cases.

### nTMS Risk Stratification

The cortical mapping verified a tumorous motor cortex infiltration in 16 cases of which 7 patients (43.8%) suffered from a new motor deficit postoperatively. In contrast, only 4 out of 50 patients (8%) without motor cortex infiltration showed a postoperative deteriorated motor status (p = .002).

**Figure 2:**
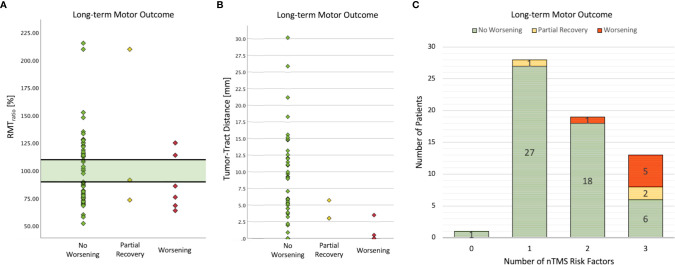
Analysis of nTMS risk factors and long-term motor outcome. No patient suffered a new permanent motor deficit when the RMT_ratio_ was between 90% and 110% **(A)**. Only one patient developed a postoperative paresis which recovered partially within 3 months. The lower the TTD was, the higher was the risk of postoperative motor deterioration **(B)**. Note that 4 patients in the worsening group had a TTD of 0 mm. Thus, in addition to the recently proposed safe TTD of 8mm, the entire nTMS risk stratification was confirmed in this cohort **(C)**.

Descriptive statistics of the nTMS parameters (RMT, MEP latency, MEP amplitude) are outlined in **Supplementary 1**. Patients with a worse motor outcome had a higher preoperative RMT_tumor_ (worsening: 73.89 V/m, SD: 30.143 vs. no worsening: 62.27 V/m, SD: 16.743; p = .101, SMD = 0.62) and a higher RMT_healthy_ (worsening: 75.89 V/m, SD: 23.513 vs. 63.90, SD: 19.438; p = .096, SMD = 0.60). Only one patient with an RMT_ratio_ between 90% and 110% had a worsened motor status after surgery, but this paresis recovered, whereas all other patients with a new deficit had an RMT_ratio_ <90% or >110% (**Figure 2**; sensitivity: 88.9%, specificity: 15.4%).

Fiber-tracking of CST showed a median tumor-tract distance (TTD) of 4.5 mm (IQR: 10.0), and no patient suffered from a new deficit when the TTD was greater than 8 mm (short-term motor outcome: cc = .302, p = .010; long term motor outcome: cc = .289, p = .019). Interestingly, the two patients with a partial recovery had a low TTD < 8mm but no tumorous infiltration of the CST (**Figure 2**). The proportion of patients with a GTR was higher in the group with TTD > 8mm (81.8%) than in the group with TTD 1-8mm (69.2%) and in the group with TTD = 0mm (44.4%) (cc = .296, p = .044).

The number of patients with a new motor deficit depending on the number of nTMS risk factors is shown in **Figure 2**. The more risk factors (motor cortex infiltration, TTD ≤ 8mm, RMT_ratio_ <90%/>110%) were detected, the higher was the risk for a worse motor outcome (cc = .506, p <.001). Postoperative imaging revealed ischemia within the motor cortex in 2 patients (3%) and within the CST in another 2 patients (3%). Subcortical ischemia resulted in permanent new motor deficits which was not the case with cortical ischemia.

The distribution of FA_avg_, FA_tumor_, ADC_avg_, and ADC_tumor_ are shown in **Supplementary 2**. Lower FA_avg_ values (worsening: mean: 0.38 (SD: 0.09), no worsening mean: 0.47 (SD: 0.07), p = .008, SMD = 1.07) and higher ADC_tumor_ (worsening: mean: 12.47 * 10^-4^ (SD: 7.99 * 10^-4^), no worsening mean: 8.91 * 10^-4^ (SD: 6.93 * 10^-4^) p = .045, SMD = 0.48) were associated with a deteriorated postoperative motor status.

### IOM

An overview of the used IOM techniques is presented in **Table 2**. Only one patient (2.9%) of 34 with stable MEP amplitude suffered from a new motor deficit postoperatively (p = .001, cc = .403) and recovered in long-term motor outcome (p = .003, cc = .408). An irreversible MEP amplitude decrease resulted in a new motor deficit postoperatively in the majority of cases (**Table 2**, p = .003, cc = .544), however patients with a decrease ≤50% of the baseline MEP amplitude were more likely to have a recovery in long-term motor outcome (**Table 2**, p = .011, cc = .601). No patient with a completely reversible MEP amplitude alteration showed postoperative motor worsening.

**Table 2:** Evaluation of the IOM.

	Short-Term Motor Outcome			Long-Term Motor Outcome				Extent of Resection		
	n = 66	No Worsening	Worsening	n = 61	No Worsening	Partial Recovery	Persistent Worsening	GTR	STR	PR
**MEP Monitoring**	61 (92.4%)	50 (82%)	11 (18%)	57 (93.4%)	48 (84.2%)	3 (5.3%)	6 (10.5%)	42 (68.9%)	16 (26.2%)	3 (4.9%)
MEP amplitude stable	34 (55.7%)	33 (97.1%)	1 (2.9%)	34 (59.6%)	33 (97.1%)	1 (2.9%)	0	27 (79.4%)	6 (17.6%)	1 (2.9%)
MEP amplitude alteration	27 (44.3%)	17 (63%)	10 (37%)	23 (40.4%)	15 (65.2%)	2 (8.7%)	6 (26.1%)	15 (55.6%)	10 (37%)	2 (7.4%)
completely reversible	11 (40.7%)	11 (100%)	0	10 (43.5%)	10 (100%)	0	0	7 (63.6%)	4 (36%)	0
irreversible + ≤50% decrease	9 (33.3%)	4 (44.5%)	5 (55.6%)	9 (39.1%)	4 (44.4%)	2 (22.2%)	3 (33.3%)	7 (77.8%)	2 (22.2%)	0
irreversible + >50% decrease	7 (25.9%)	2 (28.6%)	5 (71.4%)	4 (17.4%)	1 (25%)	0	3 (75%)	1 (14.3%)	4 (57.1%)	2 (28.6%)
**SCS Monitoring**	53 (80.3%)	44 (83%)	9 (17%)	48 (78.7%)	41 (85.4%)	3 (6.3%)	4 (8.3%)	33 (62.3%)	19 (35.8%)	1 (1.9%)
≤4mA	13 (24.5%)	7 (53.8%)	6 (46.2%)	11 (22.9%)	6 (54.5%)	2 (18.2%)	3 (27.3%)	7 (53.8%)	6 (46.2%)	0
5-7mA	24 (45.3%)	21 (87.5%)	3 (12.5%)	22 (45.8%)	20 (90.9%)	1 (4.5%)	1 (4.5%)	15 (62.5%)	8 (33.3%)	1 (4.2%)
>7mA	16 (30.2%)	16 (100%)	0	15 (31.3%)	15 (100%)	0	0	11 (68.8%)	5 (31.3%)	0
**SSEP Monitoring**	29 (43.9%)	24 (82.8%)	5 (17.2%)	26 (42.6%)	22 (84.6%)	1 (3.8%)	3 (11.5%)	21 (72.4%)	7 (24.1%)	1 (3.4%)
SSEP amplitude reversible decrease	25 (86.2%)	21 (84%)	4 (16%)	23 (88.5%)	20 (87%)	1 (4.3%)	2 (8.7%)	18 (72%)	6 (24%)	1 (4%)
SSEP amplitude irreversible decrease	4 (13.8%)	3 (75%)	1 (25%)	3 (11.5%)	2 (66.7%)	0	1 (33.3%)	3 (75%)	1 (25%)	0

MEP, motor evoked potentials; SCS, subcortical stimulation; SSEP, somatosensory evoked potentials; GTR, gross total resection; STR, subtotal resection; PR, partial resection.

Patients whose resection was performed at lower stimulation intensities ≤4mA during the SCS had the highest risk of suffering a new motor deficit, in contrast to an intensity ≥8mA, which was found to be safe from postoperative motor deterioration (**Table 2**, p = .003, cc = .422). In the cases in which SSEP monitoring was performed, there was no correlation with postoperative motor status (shot-term motor outcome: p = .658, cc = .082; long-term motor outcome: p = .437, cc = .245).

### Comparative Analysis of nTMS/DTI and IOM

**Figure 3:**
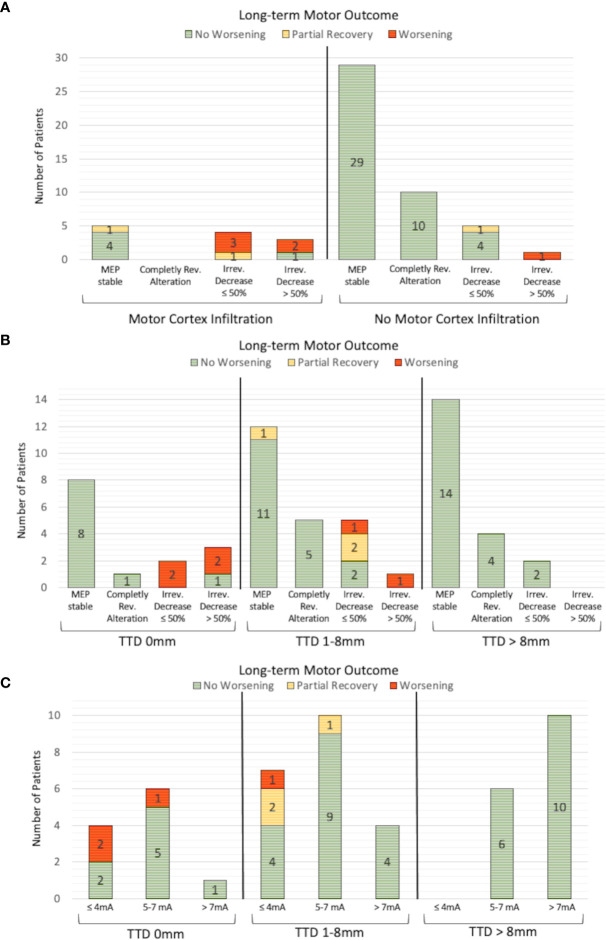
Analysis of IOM and long-term motor outcome. An irreversible MEP amplitude decrease >50% resulted in worse motor outcome in 3 of 4 patients **(A)**. An irreversible MEP amplitude decrease ≤50% resulted in a new postoperative motor deficit, particularly in patients with nTMS-verified motor cortex infiltration, that was not present in patients without motor cortex infiltration. A similar correlation could be found for the analysis of the TTD **(B)**. No patient with a TTD >8mm suffered a new motor deficit, independently of whether MEP changes were detected or not. The motor outcome of patients with an irreversible MEP decrease ≤50% is worse, especially in patients with a TTD of 0mm than in patients with a TTD between 1 and 8mm. This phenomenon is also observed for the minimum SCS intensity, where the postoperative motor outcome was worse in the group of patients with a TTD of 0 mm than in the group with a TTD between 1 and 8mm, while the same SCS intensities were used **(C)**.

In patients with preoperatively verified tumorous motor cortex infiltration, intraoperative MEP alterations occurred more frequently (**Figure 3**; 33.3% with infiltration vs. 14.7% without; p = .086, cc = .215). In 3 of 4 patients with motor cortex infiltration (75%) a permanent decrease of the MEP amplitude ≤50% resulted in a permanent deficit and in one patient (25%) in a transient deficit. In contrast, no postoperative deterioration occurred in 4 of 5 patients (80%) without verified motor cortex infiltration, although MEP amplitude was also decreased by ≤ 50%. One patient (20%) had a new motor deficit which recovered partially.

A subgroup analysis of patients with TTD >8mm revealed that MEP amplitude alterations were detected in 6 of 27 patients (22.2%), 2 of whom had an irreversible MEP amplitude decrease ≤50%, although no patient within this group deteriorated (**Figure 3**). An irreversible MEP decrease >50% was only found in the patients with a TTD ≤8mm. In a further analysis of patients with an irreversible MEP amplitude decrease ≤50%, two patients with a TTD of 0mm (100%) suffered a permanent motor deficit, whereas 1 of 5 patients (20%) with a TTD between 0 and 8mm had a new persistent motor deficit, 2 (40%) had a new paresis with partial recovery within 3 months, and 2 (40%) had no postoperative motor deterioration (**Figure 3**).

The TTD measured preoperatively and the minimum used intensity of intraoperative SCS were significantly correlated (Pearson’s r = 0.5; p <.001). An SCS intensity ≤4mA and a TTD of 0mm resulted in a permanent deficit in 2 of 4 patients (50%). In contrast, a TTD between 0 and 8mm led to a new permanent paresis in 1 of 7 patients (14.3%) and to a postoperative deterioration in 2 of 7 patients (28.6%) which partially recovered within 3 months (**Figure 3**).

Neither the RMT_tumor/_RMT_healthy_ nor the RMT_ratio_ were associated with specific findings of the IOM.

## Discussion

### Main Finding of the Study

**Figure 4:**
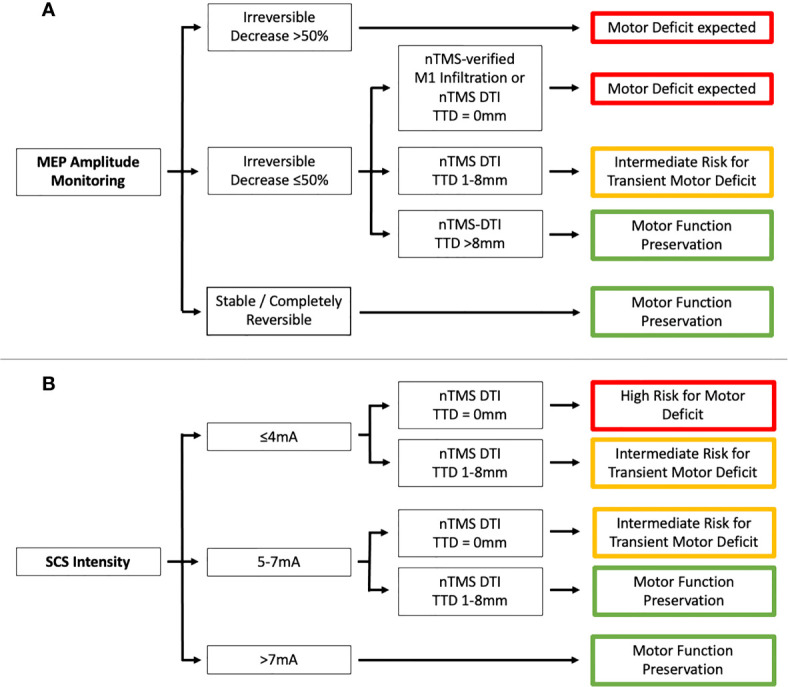
Flowchart showing the association between preoperative nTMS assessment (motor cortex [M1] infiltration and tumor-tract distance [TTD]) and IOM (**A** - MEP amplitude monitoring and **B** - SCS intensity).

To the best of our knowledge, this is the first study to analyze findings from nTMS motor mapping and DTI fiber tracking and IOM with respect to the short- and long-term motor outcome, which we have illustrated in a flow chart (**Figure 4**). The motor outcome of patients with an irreversible MEP amplitude decrease ≤50% or used SCS intensities ≤7mA depends on the nTMS risk stratification. The more risk factors (motor cortex infiltration, TTD ≤8mm, RMT_ratio_ <90%/>110%) were found, the higher was the risk for a new motor deficit. An irreversible MEP amplitude decrease >50% was always associated with worse motor outcome whereas patients with reversible MEP amplitude alterations and used SCS intensities >7mA always showed preserved motor function. The same was true when the TTD was >8mm as described before by our group (14).

### Preoperative Assessment by nTMS and DTI

The use of nTMS data for DTI fiber tracking of CST proved to be a superior technique, as peritumoral tracts in particular could be visualized in a user-independent manner and robust to peritumoral edema (25, 28). The highly significant correlation between the TTD and the minimal SCS intensity is consistent with a previously published validation study of nTMS-derived tractography (32). Our analysis also confirmed the recent risk stratification model that no new postoperative motor deficit occurred in patients with a TTD > 8 mm (14). The results of which were also confirmed externally with a similar TTD threshold of 12 mm (33, 34). In addition to the TTD, nTMS-verified tumorous infiltration of the motor cortex was also confirmed as a risk for postoperative motor deficit, which must be emphasized because tumor mass effect, peritumoral edema, and tumor-induced plasticity often confound accurate landmark-based assessment (34, 35). The nTMS mapping not only provides topographic data but also allows neurophysiological assessment whereby an unbalanced interhemispheric excitability (RMT_ratio_ <90%/>110%) was associated with worse postoperative motor outcome (14, 36). Our analysis shows RMT_ratio_ to be a sensitive (sensitivity: 88.9%) but nonspecific (specificity: 15.4%) parameter for predicting motor outcome. On the one hand, only one patient with an RMT_ratio_ between 90% and 110% suffered a motor deficit that partially recovered within 3 months. On the other hand, 44 of 52 patients (84.6%) with an impaired RMT_ratio_ <90%/>110% maintained their motor function postoperatively. Previous studies indicated that worsened motor status was associated with higher RMT_tumor_ values, a trend that was also evident in this cohort (33). The RMT_ratio_ seems to have a limited significance in this cohort because of its low specificity. However, in combination with the other risk factors, the likelihood of motor deterioration is further increased or decreased depending on the RMT_ratio_.

### IOM – Transcranial MEP Monitoring

There is evidence from a meta-analysis that resection of eloquent brain tumors should be guided by intraoperative stimulation to increase the extent of resection and reduce the incidence of new motor deficit (5). In addition, innovative techniques such as nTMS and IOM enable surgical treatment of patients whose brain tumors were previously deemed unresectable (37). A European multicenter survey raised the (still unanswered) question which stimulation parameters and which warning criteria should be used (38).

Different thresholds regarding irreversible MEP amplitude decrease and postoperative motor deterioration have been discussed (from any irreversible change to 50% decrease and up to 80% decrease), where the studies analyzed MEPs induced by both transcranial electrical stimulation and direct cortical stimulation (9, 39–41). Reversible MEP amplitude alterations were rather associated with transient motor deficits (8, 9, 41). More recently, MEP latency prolongation was less considered for motor outcome prediction because of its low sensitivity and specificity (8, 9, 31). In agreement with the literature, no patient suffered a new postoperative paresis if the MEP amplitude was stable or showed a completely reversible change, so there were no false-negative events. On the other hand, an irreversible MEP amplitude decrease >50% led to a permanent deficit in 75% of patients, which is also in accordance with the literature. Interestingly, irreversible MEP amplitude decrease ≤50% was associated with worse motor outcome in high-risk nTMS cases, which was not true for patients without motor cortex infiltration or a TTD >8 mm. Thus, the preoperative nTMS risk stratification not only correlates with MEP amplitude alterations but also provides information that can improve the interpretation of IOM findings, especially for identifying false-positive amplitude changes. This is particularly important because recent studies have reported various neurosurgical as well as anesthesiologic causes that may affect MEP amplitude and could not be distinguished from phenomena induced by direct tissue lesions (39, 42–44). To our knowledge, our analysis is the first one that can help to distinguish true-positive from false-positive MEP amplitude alterations.

### IOM – Continuous Subcortical Mapping

The usefulness and accuracy of continuous SCS for detecting and preserving the CST have been demonstrated, since MEP alterations are rather suitable for outcome prediction than as warning system (7, 45, 46). However, MEP monitoring and continuous SCS remained as standard of care to enhance safety during low stimulation thresholds (45).

Earlier studies aimed to find a safe limit for SCS intensities (e.g., 5-6mA) with which tumor resection can be performed without risk for the motor system (31, 47–49). Raabe et al. showed that SCS intensities <5mA only causes transient motor deficits in their case series – except two patients with detected ischemia on postoperative imaging who suffered a permanent motor deterioration (7). This and other studies promoted lowering stimulation intensities to 2-3mA (7, 50, 51) but the question of which patients are appropriate for this approach has yet to be answered, as lower SCS intensities were associated with a higher risk of CST injury in our study. In our patients with a tumorous subcortical infiltration of the CST (TTD = 0mm), lowering the stimulation intensity ≤4mA was associated with a higher risk of a new permanent motor deficit. This tendency was much less pronounced in the group of patients with TTD between 1 and 8mm. Thus, the minimal SCS intensity should always be adjusted to whether a GTR is realistically possible and oncologically appropriate. We observed for the first time that the risk for a new motor deficit depends on the TTD despite the same SCS intensity. Thus, the nTMS risk stratification may additionally help to optimize the tumor resection and monitoring strategy to further minimize the risk for motor deterioration.

### IOM – Somatosensory Evoked Potentials

SSEPs were used less frequently in our cohort and showed no correlation that could contribute to the improvement of motor outcome or intraoperative guidance of resection. There is one study showing very low sensitivity with low positive predictive value of SSEP monitoring in brainstem surgery, stating that incautious interpretation may lead to unnecessary termination of tumor resection (52). However, SSEPs in brain tumor surgery have been studied more in terms of identifying the motor cortex by phase reversal of the somatosensory evoked potential and not for monitoring the integrity of the motor system (53).

### Limitations

We performed a retrospective analysis of a prospectively collected cohort of patients with motor-eloquent glioma in whom the treating neurosurgeon individually decided on the exact IOM techniques. Therefore, we cannot exclude the occurrence of selection bias. Because of our very detailed analysis of motor-eloquent brain tumors and of abnormal IOM phenomena, the resulting subgroups are small, which did not permit statistical testing for group differences. On the other hand, this allowed us to provide a sound comparative analysis of nTMS and IOM findings that has not been investigated before. Multicentric, prospectively designed studies are needed to further improve the treatment algorithm of motor-eloquent tumors.

Deterministic DTI has been used in routine clinical practice to determine TTD in a validated and user-independent approach with robustness to peritumoral edema (25, 32). New techniques such as probabilistic tractography capable of visualizing areas of complex fiber architecture have been investigated, however, these approaches have not yet been established in clinical practice (54). For visualization of the CST, we used deterministic DTI as the established clinical routine. This technique has limitations, especially in the situation of crossing/kissing fibers (55).

## Conclusions

An irreversible MEP amplitude decrease >50% was associated with higher risk of developing a new postoperative paresis in all cases. The motor outcome of patients with an irreversible MEP amplitude decrease ≤50% or used SCS intensities ≤7mA depends on the nTMS risk stratification: high-risk cases (motor cortex infiltration, TTD <8mm, RMT_ratio_ <90%/>110%) had a higher risk for postoperative motor deterioration which was not the case in low-risk patients. Thus, the preoperative nTMS-based risk assessment can improve the interpretation of IOM phenomena and the adjustment of SCS stimulation intensity. These observations warrant a prospective interventional study to address the potential impact of nTMS informed IOM interpretation on clinical outcomes.

## Data Availability Statement

The datasets presented in this article are not readily available because of restrictions due to the Data Protection Act. Requests to access the datasets should be directed to TR.

## Ethics Statement

The studies involving human participants were reviewed and approved by The ethics committee of the Charité – Universitätsmedizin Berlin (#EA1/016/19). The patients/participants provided their written informed consent to participate in this study. Written informed consent was obtained from the individual(s) for the publication of any potentially identifiable images or data included in this article.

## Author Contributions

Substantial contributions to the conception or design of the work, or the acquisition, analysis or interpretation of data for the work: TR, MS, MM, PV, TP, and KF. Drafting the work or revising it critically for important intellectual content: TR and TP. Providing approval for publication of the content: TR, MS, MM, PV, TP, and KF. Agree to be accountable for all aspects of the work in ensuring that questions related to the accuracy or integrity of any part of the work are appropriately investigated and resolved: PV, TP, and KF. All authors contributed to the article and approved the submitted version.

## Funding

The authors acknowledge the support of the Cluster of Excellence Matters of Activity. Image Space Material funded by the Deutsche Forschungsgemeinschaft (DFG, German Research Foundation) under Germany´s Excellence Strategy – EXC 2025. TR is participant in the BIH-Charité Junior Digital Clinician Scientist Program funded by the Charité – Universitätsmedizin Berlin and the Berlin Institute of Health.

## Conflict of Interest

The authors declare that the research was conducted in the absence of any commercial or financial relationships that could be construed as a potential conflict of interest.

## Supplementary Material

Supplementary 1 Analysis of neurophysiological data. The distribution of the RMT stimulator output **(A)**, the RMT electric field strength **(B)**, the MEP latency **(C)** and the MEP amplitude **(D)** is shown for the tumor and the healthy hemisphere.

Supplementary 2 Analysis of the diffusion parameters. The distribution of the FA_avg_, FA_tumor_ **(A)**, ADC_avg_ and ADC_tumor_ in mm^2^/s **(B)** is shown.
