# Supplementary figures and images for: Preoperative nTMS and Intraoperative Neurophysiology - A Comparative Analysis in Patients With Motor-Eloquent Glioma
Source: Front Oncol. 2021 May 21;11:676626. doi: 10.3389/fonc.2021.676626 (PMC8175894; doi:10.3389/fonc.2021.676626)

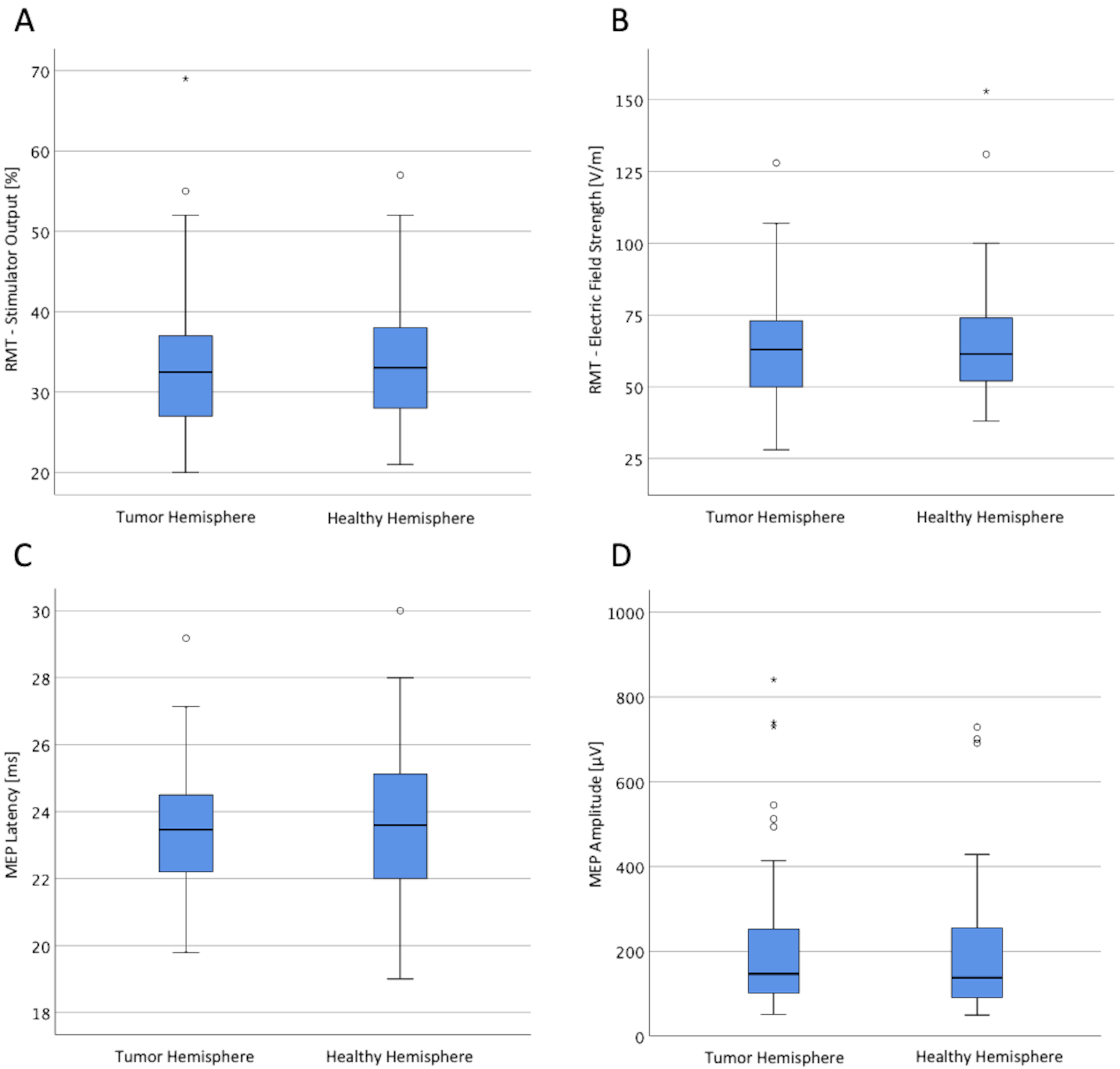

Supplement: Supplementary 1 — Analysis of neurophysiological data. The distribution of the RMT stimulator output (A), the RMT electric field strength (B), the MEP latency (C) and the MEP amplitude (D) is shown for the tumor and the healthy hemisphere. [file Image_1.tiff]

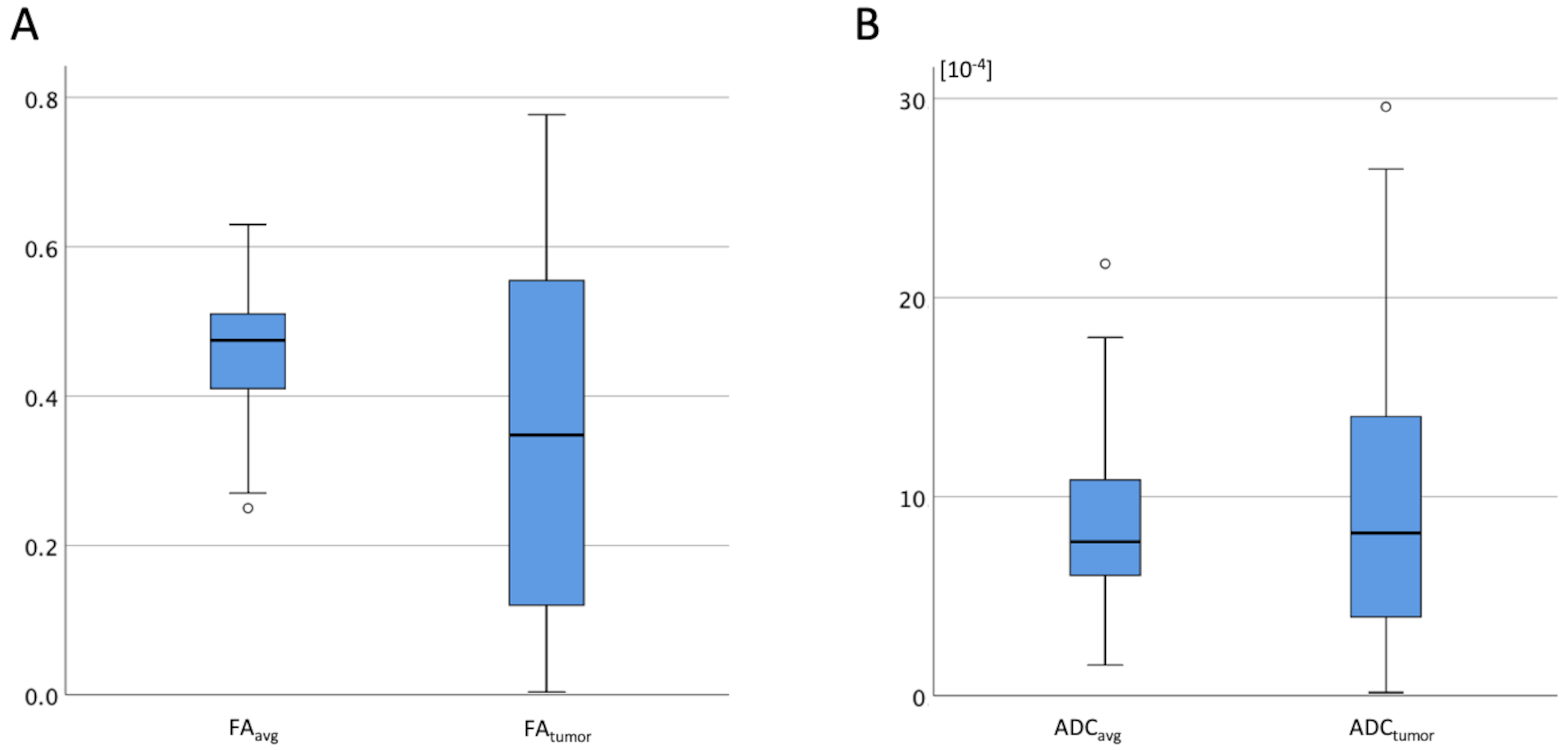

Supplement: Supplementary 2 — Analysis of the diffusion parameters. The distribution of the FAavg, FAtumor (A), ADCavg and ADCtumor in mm2/s (B) is shown. [file Image_2.tiff]
